# Multiple introductions of dengue virus strains contribute to dengue outbreaks in East Kalimantan, Indonesia, in 2015–2016

**DOI:** 10.1186/s12985-019-1202-0

**Published:** 2019-07-25

**Authors:** R. Tedjo Sasmono, Lily Pertiwi Kalalo, Suryani Trismiasih, Dionisius Denis, Benediktus Yohan, Rahma F. Hayati, Sotianingsih Haryanto

**Affiliations:** 1 Dengue Research Unit, Eijkman Institute for Molecular Biology, Ministry of Research, Technology, and Higher Education, Jl. Diponegoro 69, Jakarta, 10430 Indonesia; 2 AW Sjahranie Hospital and Department of Clinical Pathology, Faculty of Medicine, Universitas Mulawarman, Samarinda, East Kalimantan Indonesia; 3 Pertamina Hospital, Balikpapan, East Kalimantan Indonesia; 4 Siloam Hospital Jambi and Raden Mattaher Hospital, Jambi, Indonesia

**Keywords:** Dengue, Serotype, Phylogenetic, Genetic diversity, Herd immunity, Borneo, Indonesia

## Abstract

**Background:**

Dengue fever is a febrile disease caused by dengue virus (DENV), which affects people throughout the tropical and subtropical regions of the world, including Indonesia. East Kalimantan (Borneo) province suffered a dramatic increase in dengue cases in 2015 and 2016, making it the province with the second highest incidence of dengue in Indonesia. Despite this, dengue in East Kalimantan is understudied; leaving transmission dynamics of the disease in the area are mostly unknown. In this study, we investigate the factors contributing to the outbreaks in East Kalimantan.

**Methods:**

Prospective clinical and molecular virology study was conducted in two main cities in the province, namely Samarinda and Balikpapan, in 2015–2016. Patients’ clinical, hematological, and demographic data were recorded. Dengue detection and confirmation was performed using NS1-antigen and IgG/IgM antibody detection. RT-PCR was conducted to determine the serotypes of the virus. Phylogenetic analysis was performed based on envelope gene sequences.

**Results:**

Three hundred patients with suspected dengue were recruited. Among these, 132 (44%) were diagnosed with dengue by NS1 antigen and/or nucleic acid detection. The majority of the infections (60%) were primary, with dengue hemorrhagic fever (DHF) the predominant manifestation (71.9%). Serotyping detected all four DENV serotypes in 112 (37.3%) cases, with the majority of patients (58.9%) infected by DENV-3. Phylogenetic analysis based on envelope gene sequences revealed the genotypes of the viruses as DENV-1 Genotype I, DENV-2 Cosmopolitan, and DENV-3 Genotype I. Most virus strains were closely-related to strains from cities in Indonesia.

**Conclusions:**

Our observations indicate that multiple introductions of endemic DENV from surrounding cities in Indonesia, coupled with relatively low herd immunity, were likely responsible for the outbreak of the dominant viruses. The study provides information on the clinical spectrum of the disease, together with serology, viral genetics, and demographic data, which will be useful for better understanding of dengue disease in Borneo.

**Electronic supplementary material:**

The online version of this article (10.1186/s12985-019-1202-0) contains supplementary material, which is available to authorized users.

## Background

Dengue is a systemic viral infection caused by dengue virus (DENV), a member of the *Flaviviridae* family. Dengue causes significant public health problems in tropical and subtropical regions of the world, including Indonesia. There are four DENV serotypes (DENV-1, − 2, − 3, and − 4) circulating and transmitted by the *Aedes* mosquitoes vector [8, 12]. Dengue clinical manifestations vary; they can be in the form of acute febrile illness, classical dengue fever (DF) or dengue hemorrhagic fever (DHF). DHF may then develop into dengue shock syndrome (DSS) [49]. The DENV genome consists of a ~ 10.7 kb single-stranded positive-sense RNA encoding three structural (C, prM/M, E) and seven non-structural (NS1, NS2A, NS2B, NS3, NS4A, NS4B, NS5) proteins [10, 12]. The spatial and temporal distribution of dengue disease were affected by variabilities in the key elements involved, such as the mosquito vectors, humans, virus characteristics, and environmental factors [8].

The genetic diversity of DENV is characterized by the presence of four different serotypes. However, this diversity can be further extended with the presence of multiple genotypes within each serotype [13, 44, 48]. Phylogenetic analyses based on partial and/or complete genomic sequences have been used to elucidate the origins, epidemiology (genetic diversity, transmission dynamics and epidemic potential), and the forces that shape DENV molecular evolution in nature. Phylogenetic studies have improved our ability to understand and predict the emergence of DENV [48].

Indonesia has reported dengue cases since 1968 in Jakarta and Surabaya [42]. To date, dengue has affected all 34 provinces across the vast Indonesian archipelago [30]. The disease has become an annual public health problem, and many provinces in Indonesia have experienced increased cases of dengue, including the East Kalimantan province. Nationally, in 2015 East Kalimantan was the province with the second highest level of dengue morbidity in Indonesia, after Bali, with an incidence rate (IR) of 188.46/100,000 population [29]. In 2016, 10,712 cases were recorded, with an IR of 305.95/100,000 population, which was again the second highest level after Bali [30].

**Fig. 1:**
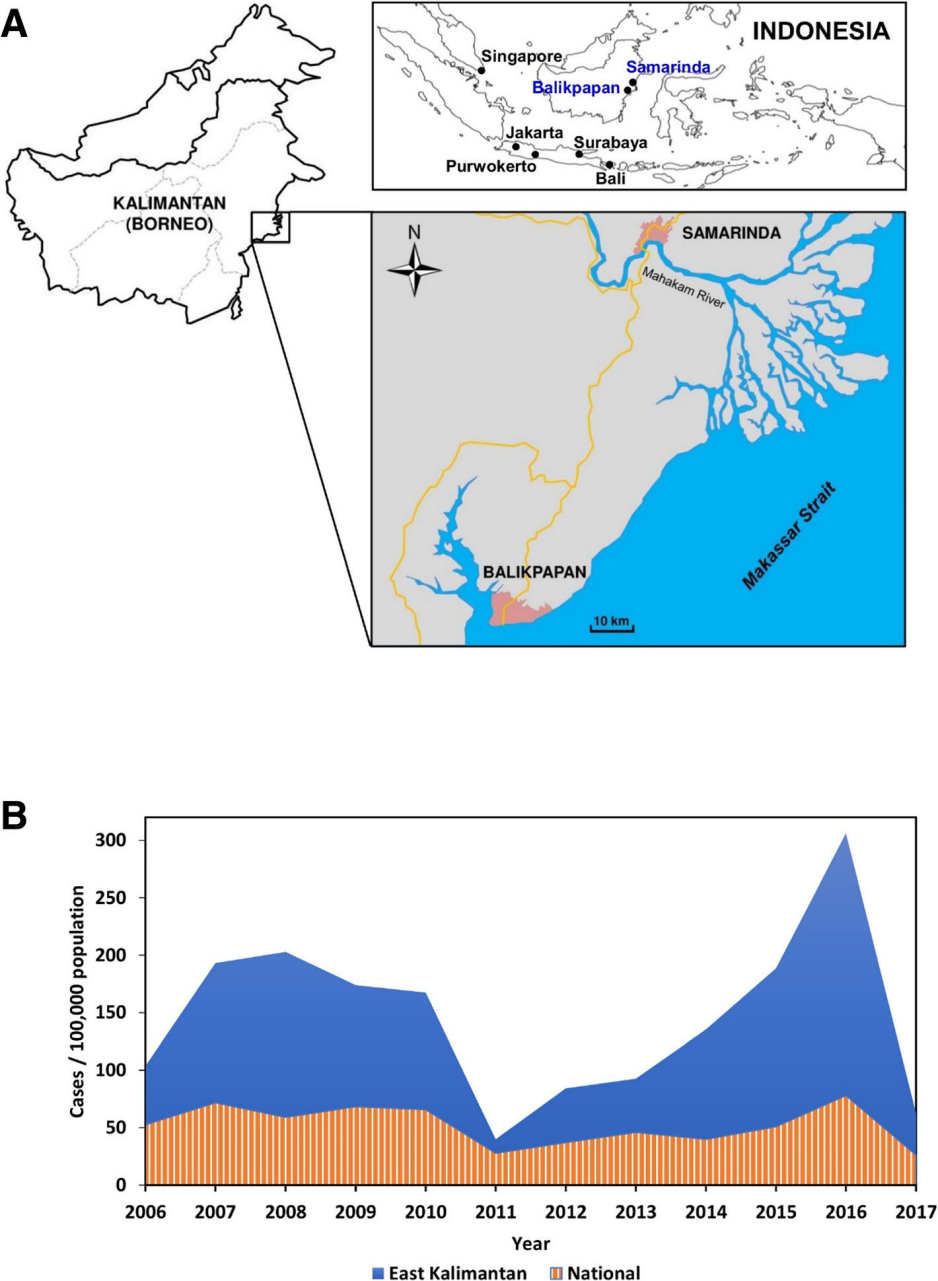
Map of study sites in East Kalimantan province, Indonesia. Samarinda and Balikpapan cities are printed in blue colour. **a**. Dengue incidence rate (IR) measured in number of cases per 100,000 populations in East Kalimantan (solid blue graph) and Indonesia (striped graph) in the last 11 years **b**

Understanding the viral origins of outbreaks is important for the implementation of public health measures that may help to avoid or mitigate future epidemics. This is particularly essential in areas where prior virological data do not exist. East Kalimantan province is located in the third largest island in the world, Borneo. The cities of Balikpapan and Samarinda (Fig. 1a) have recently experienced very high increase in dengue cases. Despite this, to our knowledge no virological surveillance has ever been conducted in the region. DENV serotype distribution data in East Kalimantan do not exist, therefore active surveillance of dengue in these cities is crucial. There is the possibility that the marked increase in cases has been caused by the introduction of a DENV genotype with high epidemic potential, as has been reported previously [18, 40].

DENV circulation is maintained in two ecologically and evolutionary distinct transmission cycles: a sylvatic cycle and a human cycle [45]. The sylvatic cycle involves non-human primates and arboreal *Aedes* mosquitoes and is known to exist in the forests of Southeast Asia and West Africa. The human cycle involves the domestic *Aedes aegypti* and peridomestic *Ae. albopictus* mosquitoes and can be found in a diverse range of environments throughout the tropics and subtropics [45]. The emergence of sylvatic DENV is one of the areas of focus of dengue research [45]. Balikpapan and Samarinda are surrounded by dense rainforests, the habitat of orangutans and other non-human primates. In fact, sylvatic DENV-1, − 2 and − 4 have been isolated in the Malaysian peninsula and Borneo [37, 45]. Therefore, it is important to assess whether sylvatic DENVs circulate and contribute to the increasing cases of dengue in East Kalimantan.

We conducted molecular surveillance with the aim of identifying and characterizing the etiological agents during dengue outbreaks in East Kalimantan. The study was conducted in 2015–2016, during which time the dengue incidence rate (IR) in East Kalimantan was almost four times higher than the national IR [29]. Symptomatic patients with suspected dengue infection were recruited in hospitals, blood samples were collected and the infecting viruses were isolated, serotyped, and their genomes sequenced to determine their genetic identity and the origin of the DENV causing the outbreaks. The clinical features, along with demographic data, were recorded and correlated with the serotype of the infecting viruses.

## Materials and methods

### Ethical considerations

The study was approved by the Research Ethics Committee of the Eijkman Institute for Molecular Biology, Jakarta, Indonesia (Ethics Approval No. 92/2015). Written informed consent was obtained from all patients and/or legal guardians for children.

### Study design, site, time, patient recruitment, and dengue data compilation

This cross-sectional study was performed in Samarinda and Balikpapan cities, East Kalimantan province. Samarinda (0.4948° S, 117.1436° E) is the capital city of the province, with a population of 812,597 in 2015, while Balikpapan (1.2379° S, 116.8529° E) is the main seaport city in the province, with a population of 615,574 in 2015. The two cities are about 120 km apart and separated by the Mahakam River (Fig. 1a). Patients suspected of having dengue were recruited at AW Sjahranie General Hospital, Samarinda, and Pertamina Hospital, Balikpapan, East Kalimantan. Patients recruitments were conducted for 6 months from November 2015 to April 2016; this period covered one monsoon and a dengue peak season in East Kalimantan. We recruited febrile patients with temperatures of ≥38 °C with less than 5 days of fever, accompanied by at least two of the clinical symptoms of dengue, i.e. headache, retro-orbital pain, myalgia, arthralgia/bone pain, and rash [49]; aged up to 80 years old; and willing to participate in the study. Exclusion criteria were fever patients with upper respiratory tract infections and/or those diagnosed as non-dengue. The patients were interviewed by a doctor or research nurse to record their demographic data and for them to provide informed consent. Upon consent, single 3 to 5 ml blood samples were taken during an acute phase. Sera were separated by centrifugation and kept frozen until further processing. Participants’ medical and relevant clinical information were recorded.

The clinical manifestation of dengue patients was determined according to WHO-SEARO guidelines [49], which employ dengue fever (DF) and dengue hemorrhagic fever (DHF) classifications, including dengue shock syndrome (DSS). Patients with unusual manifestations and severe organ involvement, such as of the liver, kidneys, brain or heart, were considered to be suffering from expanded dengue syndrome. Diagnosis of dengue fever was also made according to these guidelines, patients with clinical symptoms of dengue and who tested positive for dengue IgM and/or IgG were classified as “probable dengue”, while patients who tested positive for the dengue NS1 antigen and/or RT-PCR were categorized as “confirmed dengue” [49]. Data on the dengue fever IR in East Kalimantan and Indonesia were compiled from published reports by the Ministry of Health of the Republic of Indonesia (*Profil Kesehatan Indonesia* / Indonesia Health Profiles 2006–2016), accessible as data repository at http://www.depkes.go.id/resources/download/pusdatin/profil-kesehatan-indonesia/.

### Serology, antigen, and viral RNA detection

Acute serum samples were first tested for the DENV-encoded Non Structural Protein 1 (NS1) antigen using the NS1 Ag Rapid Test (SD Bioline, Korea) for dengue confirmation [49]. Dengue IgG and IgM were detected using Dengue Duo IgM and IgG Capture ELISA (Panbio, Alere, Waltham). This was also used to differentiate between primary and secondary dengue infection, and employed according to the manufacturer’s instructions. The blood samples were also analyzed for common blood parameters such as hemoglobin, hematocrit, erythrocyte, platelet and leucocyte count.

For DENV nucleic acid detection, RNAs were extracted directly from 200 μL of serum samples using the MagNA Pure Total Nucleic Acid extraction kit (Roche, Mannheim, Germany), and with the automated MagNA Pure LC 2.0 extraction system (Roche), following the manufacturer’s instructions. DENV detection and serotyping were performed using real-time Simplexa™ Dengue RT-PCR assay (DiaSorin, Saluggia, Italy) [39], according to the method detailed by the manufacturer.

Full length envelope (E) gene sequencing (1485 nt for DENV-1 and -2 and 1479 nt for DENV-3) was performed, as described previously [25]. Briefly, cDNA was generated from DENV RNA using Superscript III Reverse Transcriptase (Invitrogen-Thermo Scientific). Sequencing templates were PCR-amplified from cDNA using *Pfu* Turbo Polymerase (Stratagene-Agilent Technologies, USA). PCR products were purified on 0.8% agarose gel using the QIAquick gel extraction kit (Qiagen), and cycle sequencing reactions using BigDye Dideoxy Terminator kits v.3.1 (Applied Biosystems-Thermo Scientific) were performed, employing six overlapping primers for each serotype from both strands [25]. Capillary sequencing on purified DNA was performed on a 3130xl Genetic Analyzer (Applied Biosystems). SeqScape v.2.5 software (Applied Biosystems) was used to assemble the sequence reads, with manual inspection to clarify sequence ambiguities. All the E gene sequences were deposited in the GenBank repository, with accession numbers shown in Table 1.

**Table 1:** Samples with sequenced DENV E genes

No.	Sample ID	Serotype	Genotype	Age (y)	Manifestation	Accession No.
1.	BLP-011	DENV-1	I	23	DHF	MH036375
2.	BLP-031	DENV-1	I	5	DF	MH036376
3.	BLP-079	DENV-1	I	15	DF	MH036377
4.	BLP-089	DENV-1	I	16	DF	MH036378
5.	SMD-003	DENV-1	I	12	DHF	MH036379
6.	SMD-022	DENV-1	I	2	DHF	MH036380
7.	SMD-048	DENV-1	I	15	DHF	MH036381
8.	SMD-073	DENV-1	I	31	DHF	MH036382
9.	SMD-075	DENV-1	I	4	DF	MH036383
10.	SMD-079	DENV-1	I	7	DF	MH036384
11.	SMD-088	DENV-1	I	11	DHF	MH036385
12.	SMD-138	DENV-1	I	11	DHF	MH036386
13.	SMD-142	DENV-1	I	20	DHF	MH036387
14.	BLP-017	DENV-2	Cosmopolitan	22	DF	MH036388
15.	BLP-094	DENV-2	Cosmopolitan	9	DF	MH036389
16.	BLP-095	DENV-2	Cosmopolitan	7	DF	MH036390
17.	BLP-096	DENV-2	Cosmopolitan	11	DF	MH036391
18.	SMD-041	DENV-2	Cosmopolitan	3	DHF	MH036392
19.	SMD-126	DENV-2	Cosmopolitan	6	DHF	MH036393
20.	SMD-134	DENV-2	Cosmopolitan	15	DHF	MH036394
21.	BLP-004	DENV-3	I	8	DHF	MH036395
22.	BLP-008	DENV-3	I	4	DF	MH036396
23.	BLP-014	DENV-3	I	28	DHF	MH036397
24.	BLP-015	DENV-3	I	1	DHF	MH036398
25.	BLP-025	DENV-3	I	1	DF	MH036399
26.	BLP-036	DENV-3	I	1	DF	MH036400
27.	BLP-044	DENV-3	I	4	DHF	MH036401
28.	BLP-052	DENV-3	I	3	DF	MH036402
29.	BLP-082	DENV-3	I	16	DHF	MH036403
30.	BLP-124	DENV-3	I	12	DHF	MH036404
31.	BLP-131	DENV-3	I	21	DHF	MH036405
32.	SMD-024	DENV-3	I	22	DHF	MH036406
33.	SMD-031	DENV-3	I	9	DHF	MH036407
34.	SMD-032	DENV-3	I	3	DHF	MH036408
35.	SMD-037	DENV-3	I	12	DHF	MH036409
36.	SMD-045	DENV-3	I	10	DHF	MH036410
37.	SMD-058	DENV-3	I	14	DHF	MH036411
38.	SMD-092	DENV-3	I	11	DHF	MH036412
39.	SMD-107	DENV-3	I	4	DHF	MH036413
40.	SMD-115	DENV-3	I	0.2	DHF	MH036414
41.	SMD-120	DENV-3	I	12	DHF	MH036415

### DENV phylogenetic and evolutionary analysis

The phylogenetic and evolutionary analyses of DENV from East Kalimantan were made using sequence alignment and comparison with publicly available DENV sequences in the GenBank repository, as of 20 March 2018. The search query was targeted to retrieve sequences of DENV from each serotype with more than 1,400 nt. Retrieved sequences were screened to remove all non-related sequences or coding sequences of a single gene other than the E gene. Using this approach, the initial screening yielded taxon numbers of 5391, 4240, and 2342, for DENV-1, −2, and − 3, respectively. The relatedness of East Kalimantan isolates with other isolates worldwide was sought by performing rapid multiple sequence alignment using MAFFT and FastTree 2 [17, 36]. The strains that were closely related to the East Kalimantan isolates were selected to generate a dataset for each serotype. For clarity of the tree view, we selected only 60 of the most closely related strains. Each dataset was screened for a possible recombination event using RDP4 software [27]. The resulting alignment of 1,485 nt (1,479 nt for DENV-3) was then used in a robust phylogenetic and evolutionary analysis using the Bayesian Markov Chain Monte Carlo (MCMC) algorithm, as implemented in BEAST v.1.8.4 [4, 5].

The dataset for each serotype was uploaded to the BEAUti v.1.8.4 graphical interface, with the isolation year used for the calibration of each taxon. A phylogenetic tree was inferred, based on selection of the statistical model for likelihood calculation, using jModelTest v.2.1.4 [3], while the TrN (TN93) method with four Gamma parameters (G_4_) was selected based on the Bayesian Information Criterion (BIC) results. Molecular clock measurement was set using a relaxed uncorrelated lognormal molecular clock and Bayesian skyline prior. The analysis was set to generate 100 million chains sampled for every 1000 chains. The initial estimated evolutionary rate was set at 7.6 × 10^− 4^ substitutions per site per year [2]. The MCMC trace was analyzed using Tracer v.1.5.0 to monitor the adequate Effective Sampling Size (ESS) for all the parameters after 10% burn-in and a maximum clade credibility (MCC) tree was created using TreeAnnotator v.1.8.4 and visualized using FigTree v.1.4.0. The evolutionary parameters were estimated as a median number with 95% Highest Posterior Density (HPD). The classification of the genotypes in each serotype was based on classifications by Goncalvez et al. [7], Twiddy et al. [44], and Lanciotti et al. [23], for DENV-1, − 2, and − 3, respectively.

## Results

### Dengue cases and temporal data in East Kalimantan

The dengue surveillance study was conducted in two hospitals in two cities in East Kalimantan, namely Samarinda and Balikpapan (Fig. 1a). The study was conducted in 2015–2016, a period when data from the Ministry of Health of the Republic of Indonesia show a marked increase in the incidence rate (Fig. 1b). The dengue incidence rate in East Kalimantan in 2016 was nearly twice that of 2015, with figures consistently almost four times higher than the national incidence rates. During the sampling period, a total of 300 serum samples were collected, with equal numbers for both cities. The sample number was sufficient to meet the minimum sample size required for statistical analysis.

### Patient demographics, diagnosis, and clinical characteristics

**Fig. 2:**
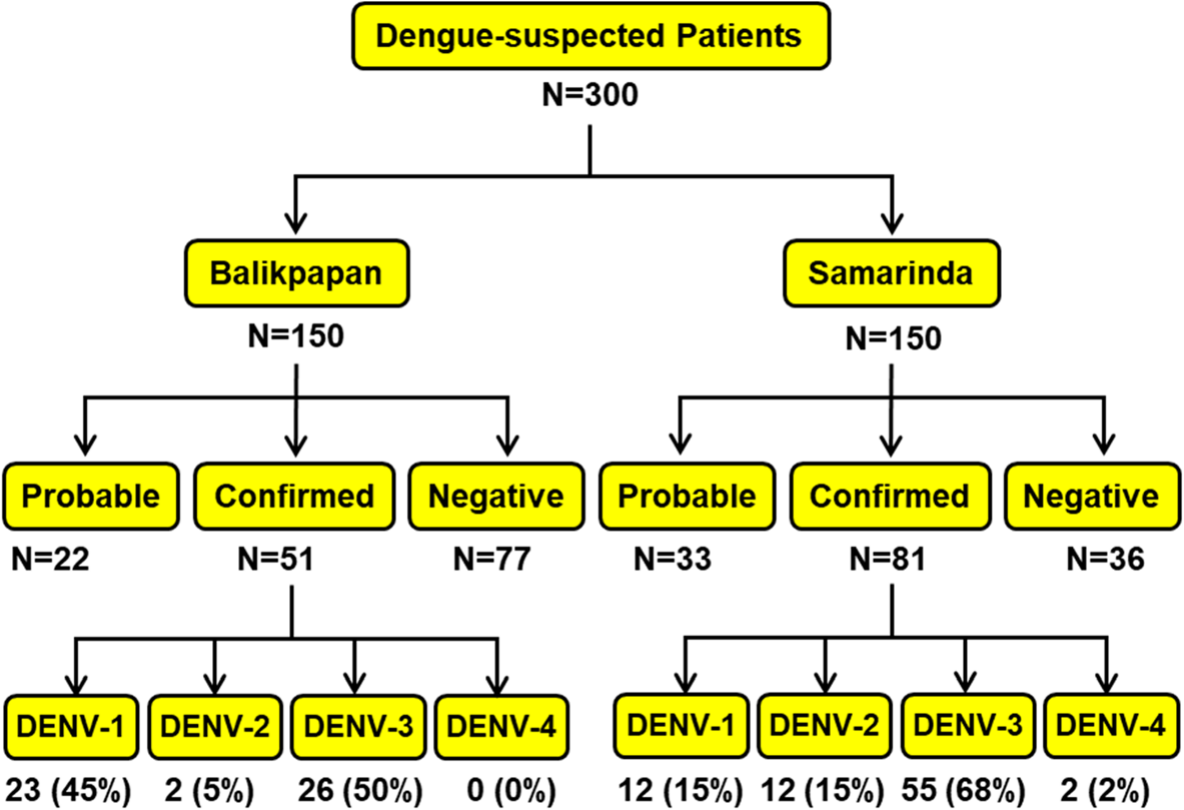
Study flow chart for dengue patient recruitment, diagnosis, and DENV serotype distribution in Samarinda and Balikpapan cities

Three hundred dengue-suspected patients (150 patients from each city) were recruited and gave informed consents. They were diagnosed according to WHO-SEARO guidelines [49]. Detection using NS1 rapid tests and/or RT-PCR was positive in 132 of them, and they were classified as confirmed dengue patients. Based on the positivity of dengue IgM and/or IgG ELISA, in total 55 patients were categorized as probable dengue patients (Fig. 2). Of the 132 dengue-confirmed patients, primary infection was detected in 60% of cases, with secondary infection in 40%.

**Fig. 3:**
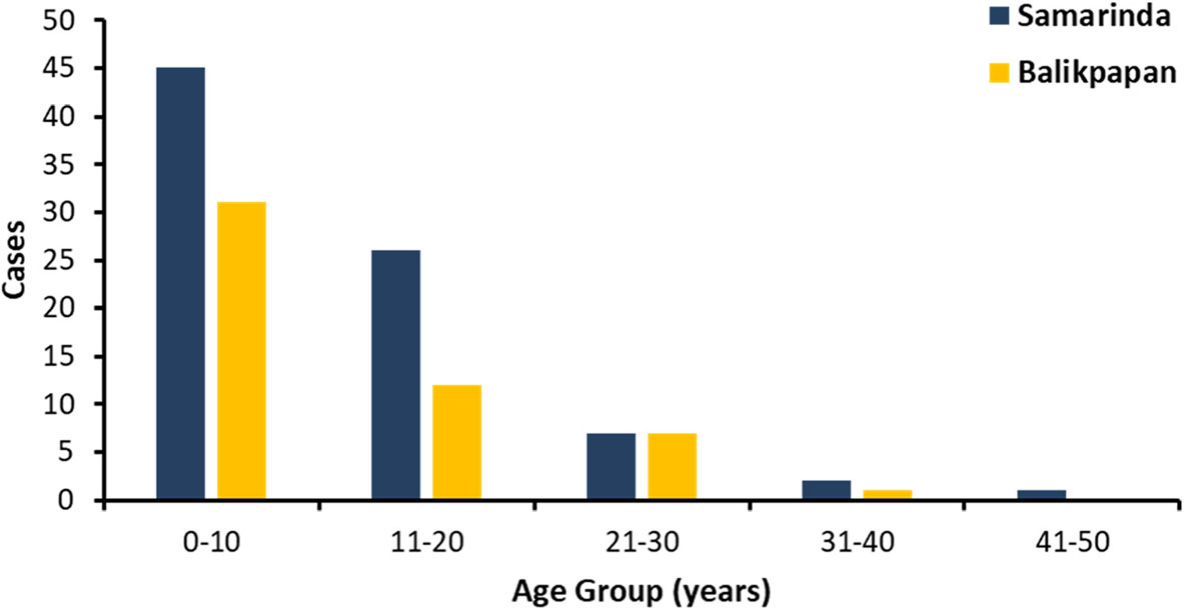
Age of confirmed dengue patients in Samarinda and Balikpapan

Among the 132 dengue-confirmed patients, the majority (107, or 81.0%) were children (≤15 y.o.) and 25 (19.0%) were adolescents and adults (Fig. 3). Patient ages ranged from 2 months up to 43 years. Eighty one (61.4%) were male and 51 (38.6%) female (a female to male ratio of 1: 1.58). We observed 35 patients (26.6%) to be DF, 95 (71.9%) manifested as DHF, and two patients as DSS (Table 2). We correlate the severity of the disease with infection status (i.e. primary vs secondary infection) and infecting serotypes, but no significant difference was observed (Additional file 1: Table S1). In addition to fever, most patients experienced malaise, nausea, headache, loss of appetite, and stomachache, while rash, arthralgia, and myalgia were less observed (Table 2). No fatalities were observed in our patient cohorts.

**Table 2:** Clinical symptoms of confirmed dengue patients

Parameters	N (%)
Severity	
DF	35 (26.5)
DHF	95 (72.0)
DSS	2 (1.5)
Clinical symptoms	
Fever	132 (100)
Malaise	98 (74.2)
Nausea	96 (72.7)
Headache	94 (71.2)
Loss of appetite	91 (68.9)
Stomachache	90 (68.2)
Positive Tourniquet test	65 (49.2)
Leucopenia	52 (39.4)
Rash	49 (37.1)
Anxiousness	47 (35.6)
Arthralgia	44 (33.3)
Myalgia	43 (32.5)
Drowsiness	31 (23.5)
Retro-orbital pain	23 (17.4)
Mucosal bleeding	9 (6.8)
Allergy	8 (6.0)
Liver enlargement > 2 cm	4 (3.0)
Fluid accumulation and breathing difficulty	2 (1.5)
Altered consciousness	1 (0.8)
Heart problem	0 (0.0)

### DENV serotype distribution

Dengue RT-PCR screening was performed in all 300 samples and DENV was successfully detected in 132 (44.0%) patients, comprising all four serotypes. An exception was DENV-4, which was not detected in Balikpapan. The vast majority of the dengue cases were caused by DENV-3 infection, accounting for 61.4% of the total sample, followed by DENV-1 (26.5%) and DENV-2 (10.6%). DENV-4 was detected only in two patients (1.5%) from Samarinda. The detailed number and percentages of DENV serotypes in each city are shown in Fig. 2.

### Phylogenetic and evolutionary analyses

**Fig. 4:**
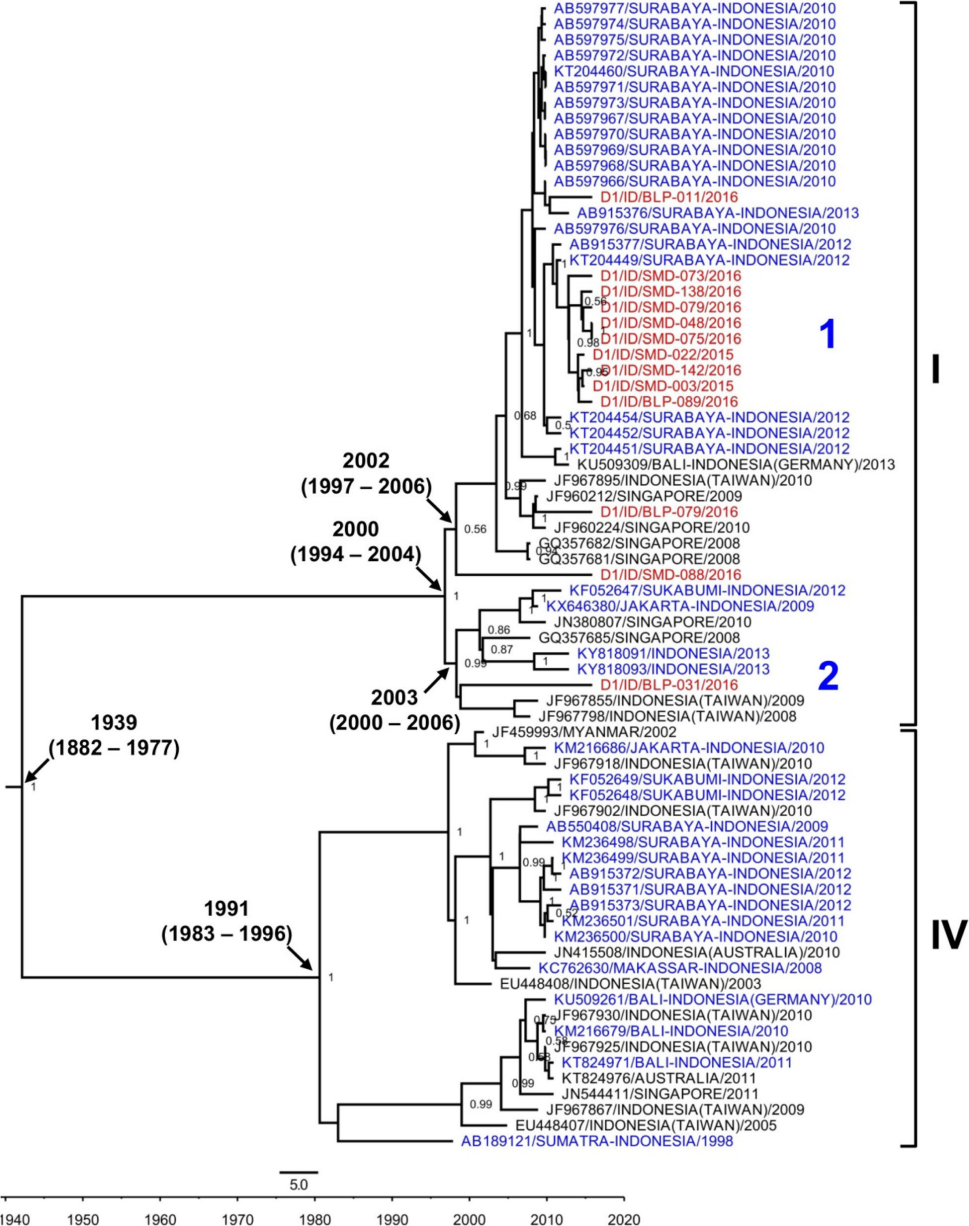
Phylogeny of DENV-1 genotype I and IV strains generated by BEAST Bayesian inference method with TrN + G evolution model calculated using E gene sequences. The red labels indicate isolates from East Kalimantan while blue labels indicate strains from other cities in Indonesia. The number in the node indicates the posterior probability of that particular cluster, with values higher than 0.5 shown. Arabic numbers depict DENV lineages

**Fig. 5:**
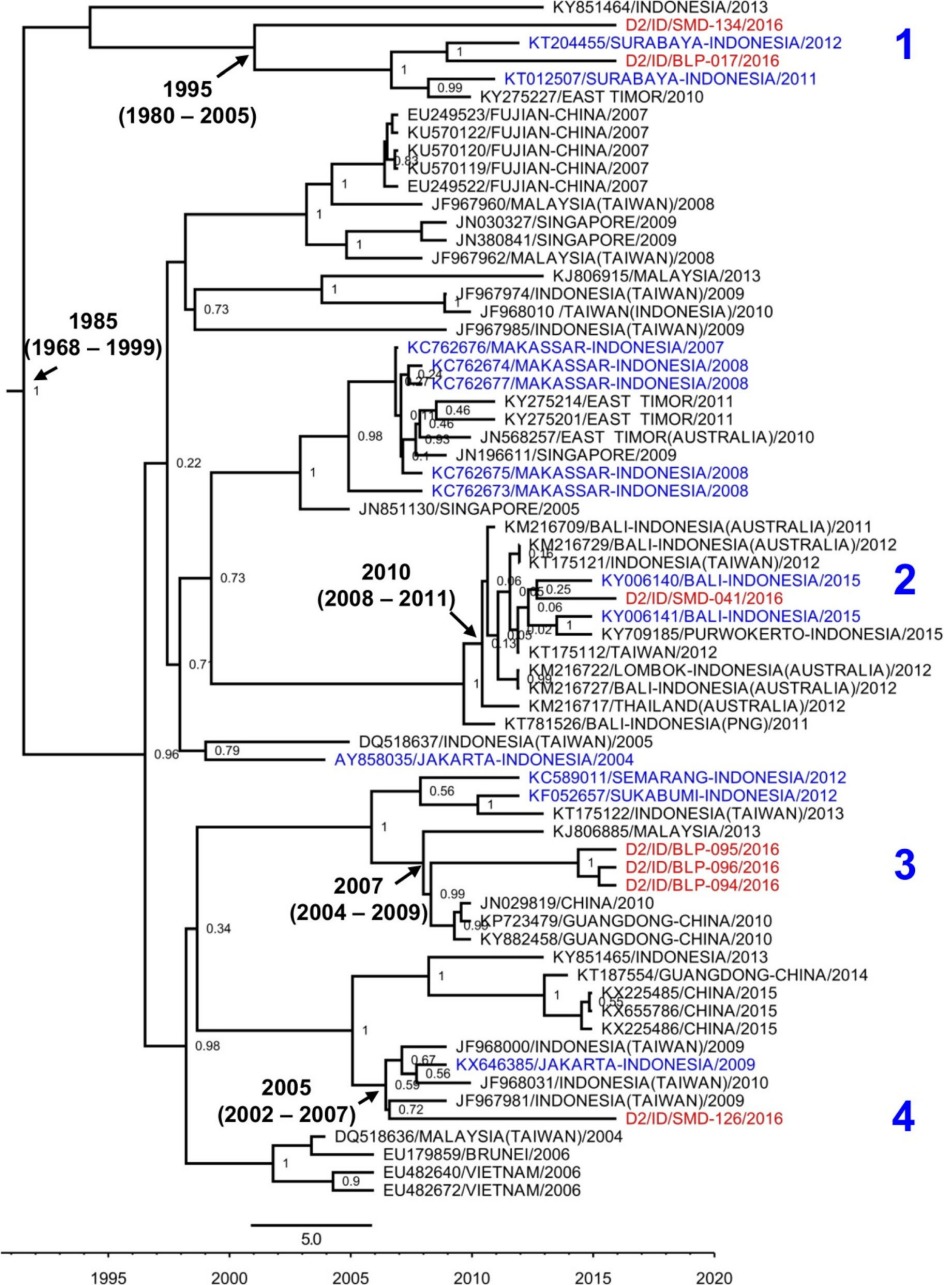
Phylogeny of DENV-2 Cosmopolitan genotype strains generated by BEAST Bayesian inference method with TrN + G evolution model calculated using E gene sequences. The red labels indicate isolates from East Kalimantan while blue labels indicate strains from other cities in Indonesia. The number in the node indicates the posterior probability of that particular cluster, with values higher than 0.5 shown. Arabic numbers depict DENV lineages

**Fig. 6:**
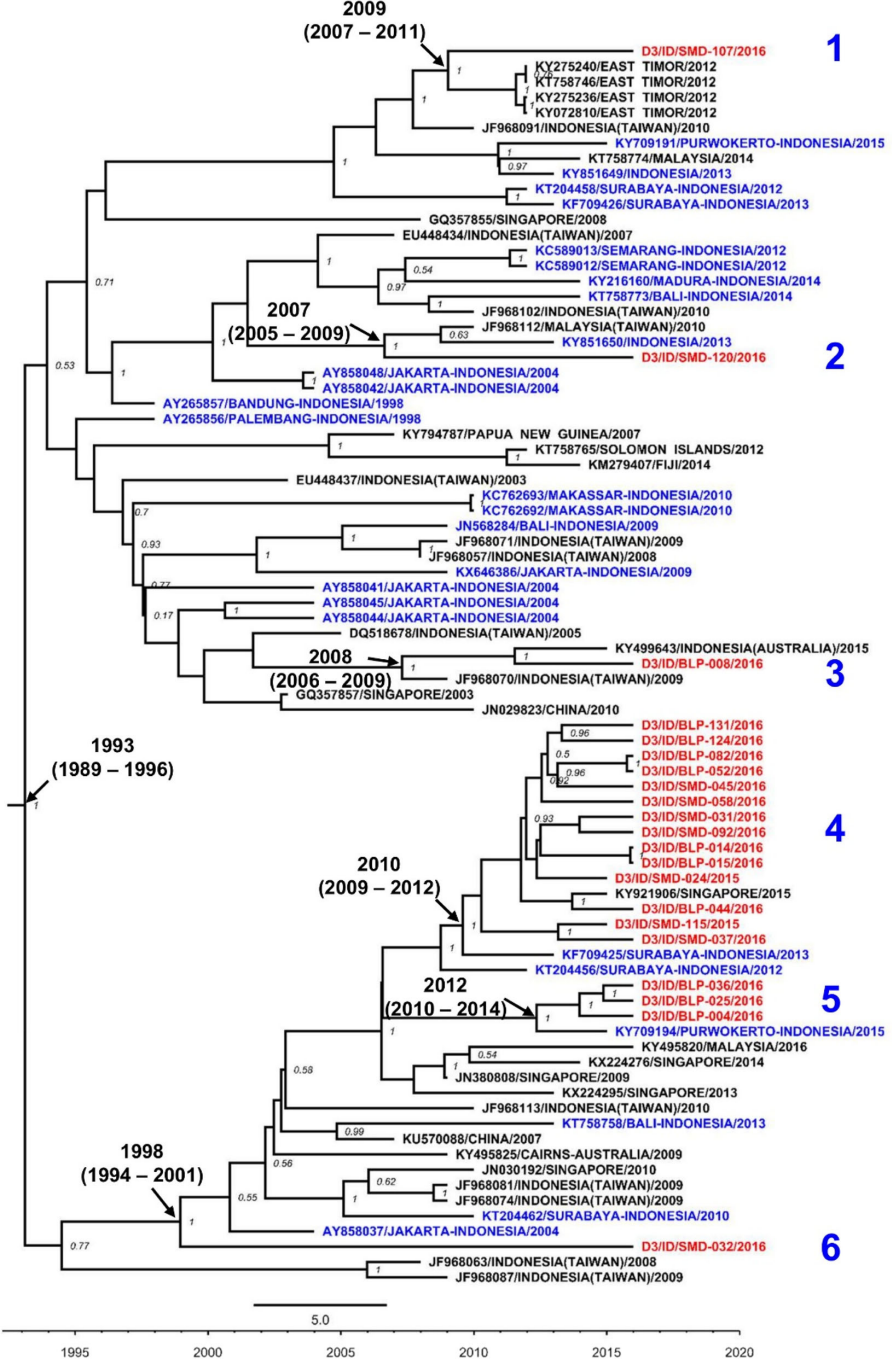
Phylogeny of DENV-3 genotype I strains generated by BEAST Bayesian inference method with TrN + G evolution model calculated using E gene sequences. The red labels indicate isolates from East Kalimantan while blue labels indicate strains from other cities in Indonesia. The number in the node indicates the posterior probability of that particular cluster, with values higher than 0.5 shown. Arabic numbers depict DENV lineages

The Bayesian evolutionary analysis generated trees of DENV-1, DENV-2, and DENV-3, with parameters as described in Additional file 2: Table S2. The overall mean evolutionary rate of each dataset was in the range of 5.1–11.5 × 10^− 4^ substitution/site/year. The age of the DENV-1 tree was estimated to date back to circa 1939, while the DENV-2 and DENV-3 trees were dated ca. 1985 and 1993 respectively (Figs. 4, 5 and 6).

A total of 13 DENV-1 sequences were successfully obtained. All of the sequences were grouped together into Genotype I (Fig. 4). The molecular clock analysis estimated that this genotype emerged ca. 2000, while the older Genotype IV, which was not detected in East Kalimantan, dated back to circa 1991 (Fig. 4). Within Genotype I, the isolates were further grouped into two major clades/lineages. We revealed the presence of a cluster of sequences closely related to strains from Surabaya, East Java, Indonesia [19] and Singapore [24] (lineage 1). One isolate from Balikpapan was found to be closely related to strains of imported DENV cases in Taiwan, which originated from Indonesia [15] (lineage 2). Both clades dated back to circa 2002–2003.

The DENV-2 isolates from East Kalimantan were grouped into the commonly found Cosmopolitan genotype. These 7 isolates were further divided into 4 distinct clades (Fig. 5). Two isolates in clade 1 were closely related to strains from Surabaya isolated in 2011 [20] and 2012 [47]. Together, these isolates may have been in circulation since circa 1995. One DENV-2 isolate from Samarinda was grouped in lineage 2, together with strains from other cities in Indonesia, and was closely related to a strain from Bali [28]. Within lineage 3, a total of 3 isolates from Balikpapan were grouped together and related to strains from Guangdong, China. Finally, an isolate from Samarinda was closely related to strains from imported dengue cases in Taiwan [15] (lineage 4).

DENV-3 was the most prevalent DENV serotype during the outbreaks. We successfully sequenced 21 isolates and constructed a phylogenetic tree, together with previously published DENV E gene sequences. All DENV-3 isolates from East Kalimantan were grouped into the single Genotype I (Fig. 6). Although all belonged to one genotype, considerable genetic diversity was observed. Within the tree, two major clades were generated, in which the East Kalimantan DENV was dispersed into six different lineages (Fig. 6). Three isolates from Samarinda were grouped together with other strains and estimated to have been circulating since 2007–2009 (lineages 1, 2 and 3). The isolates were grouped together with strains isolated from Australian soldiers returning from East Timor (now Timor Leste) and strain of dengue cases imported to Taiwan from Indonesia and Malaysia [15]. The majority of DENV-3 isolates from East Kalimantan were clustered in lineage 4. The isolates were closely related to strains from other cities in Indonesia, i.e. Surabaya [21, 47] and Purwokerto [22], and also a strain from Singapore. An interesting finding is shown by one isolate, SMD-032 (lineage 6), which generated a basal node with the most recent common ancestors (TMRCA) analysis revealed that this isolate may have been circulating in East Kalimantan since circa 1998 (Fig. 6).

Unfortunately, we failed to generate sequence of DENV-4 samples from Samarinda.

## Discussion

In this study, we have reported the characteristics of dengue outbreaks in East Kalimantan in 2015–2016, describing the virological, serological and clinical findings. DENV transmission exhibits an interannual periodicity, consisting of outbreaks every 3–5-years in peak transmission, regardless of the serotype, in many populations [8]. IR data (Fig. 1b) show the occurrence of two major outbreaks in East Kalimantan within the last decade, in 2007–2008 and the 2015–2016. The data suggest that periodic outbreaks in East Kalimantan occur once every 7–8 years, a period which is quite similar to outbreak periods in surrounding countries. A study in Thailand reported a dengue peak transmission periodicity of 7–9 years [32], while in Singapore the periodicity of transmission was reported to be 5–6 years, regardless of serotype [34]. In 2015–2016 outbreaks, we demonstrated hyperendemic DENV transmission in East Kalimantan, with all four serotypes co-circulating in the area, and with DENV-3 being the predominant serotype responsible. DENV-1 and -2 were also detected in both cities, while DENV-4 was only found in Samarinda. Overall, our study provides the first data on the 7–8 years outbreak period and the DENV serotypes circulating in East Kalimantan, which will be useful for future virological surveillance and outbreak prediction, including the magnitude, timing and location of future dengue epidemics.

We observed the predominance of different serotypes in East Kalimantan compared to other cities in Indonesia, such as in Makassar, Jambi, Surabaya and Jakarta [11, 25, 40, 47]. The serotype distribution patterns between the cities apparently differ from each other. The predominance of DENV-3 in East Kalimantan is more similar to that observed in Bali in 2015 [28]. Reports have proposed the phenomenon of serotype replacement in places in Indonesia where DENV-1 has become the predominant serotype [6, 11, 40, 47, 50], but this was not the case in East Kalimantan. As there is no previous data on DENV serotypes in Kalimantan, we do not know whether DENV-3 replaced others previously predominant in the area during the 2007–2008 outbreaks.

The clinical features of dengue infection in East Kalimantan were characterized by the presence of more DHF compared to DF (Table 2). This was distinct from our finding in Jambi, where more DF was observed [11]. We correlate the disease severity with infection status and infecting serotypes, however, no significant correlation was observed (Additional file 1: Table S1). The fact that DHF is more prominent is somewhat unusual given the higher proportion of primary infections in East Kalimantan, since it is known that secondary infection is a risk factor for increased severity [9]. However, the predominance of DENV-3 in the East Kalimantan may be the cause of the higher number of DHF. This is in line with previous report that primary infection with DENV-3 in Southeast Asia increased the risk of severe dengue infections [41]. In terms of clinical symptoms, the most common symptoms were fever, followed by malaise, nausea, headache, and loss of appetite. As expected, we found that DHF cases were more likely to have lower platelet counts compared to DF cases (Additional file 1: Table S1). Altogether, these observations are consistent with WHO-SEARO dengue clinical features [49].

The sharp increase in dengue cases in East Kalimantan warrants investigation. One possible cause is the immunity level of the local population. Because of the low herd immunity of the population, sudden increases in cases tend to occur more frequently and with even greater intensity [34]. We suspect that low herd immunity in East Kalimantan is partly responsible for the sharp increase of dengue cases. Our data show that the proportion of patients with previous DENV infection (determined as secondary infection) was only 40%. This figure is lower than that observed in Java, where dengue is hyperendemic; for example, in Semarang and Jakarta, where 77 and 81.3% of patients displayed secondary infection, respectively [6, 25]. In addition, our dengue IgG seroprevalence study conducted in 2014 on children under 18 years of age in Samarinda, which was performed as part of dengue serological surveillance in Indonesia [35], also revealed relatively low dengue immunity in East Kalimantan (data not shown), compared to national seroprevalence figure of 69.4% [35]. The 40% seroprevalence in East Kalimantan is lower than the ≈50–85%% threshold predicted to provide herd immunity that can stop arbovirus outbreaks [1, 16, 38]. Overall, these data suggest that relatively low herd immunity in East Kalimantan people may contribute to the outbreaks of dengue in 2015–2016.

Molecular surveillance of circulating DENV strains is vital in understanding the origins, genetic diversity, transmission dynamics and epidemic potentials of dengue. Particular emphasis should be directed to areas where no, or limited, virological data are available, such as areas in Kalimantan. The introduction of a new DENV serotype/genotype/lineage into a region may result in an increase in the number of dengue cases and the severity of clinical symptoms [7, 18]. Our phylogenetic analysis results provide information on the genotype and origin of the viruses circulating in the area. The predominant serotype DENV-3 was classified as Genotype I. This genotype has been shown to be the cause of the four epidemics in the region in the past [33]. In-depth analysis of East Kalimantan isolates, together with globally isolated DENV-3 viruses (*N* = 2342), shows the clustering of East Kalimantan isolates with local, endemic Indonesian strains, rather than with DENV from surrounding countries. Multiple introductions of DENV-3 from various regions in Indonesia, and not the re-emergence of local endemic DENV-3 (represented by an old isolate SMD-032 in Fig. 6) from East Kalimantan, are most likely to be responsible for the outbreak. In terms of the geographic origins of the DENV-3 strains related to the outbreak, we observed the close-relatedness of many East Kalimantan isolates with strains from Surabaya, East Java, which implies that the DENV strains originated from this city. East Java is Indonesia’s second most populous province and is geographically quite close to East Kalimantan. The extensive shipping lines and air transport between Surabaya and cities in East Kalimantan clearly facilitate the migration flow from East Java to East Kalimantan. Indeed, East Kalimantan is the third migration destination for East Java residents after Jakarta and West Java [43].

The mean evolutionary rates of each DENV serotype from East Kalimantan were relatively similar, as shown by the overlapping 95% HPD estimates (Additional file 2: Table S2), although DENV-3 showed a relatively higher evolutionary rate compared to the other serotypes. Overall, these estimates were slightly higher than numbers reported in a previous report [2]. Higher mutation rates might indicate that this particular lineage was spreading relatively quickly, as in outbreak situation. In terms of DENV genetic identity between Samarinda and Balikpapan, our analysis shows that there is no clear genetic divergence in the DENV isolated from the two cities, as shown by the clustering of isolates from both cities in the same clades, suggesting that the viruses in circulation most likely originated from a single place, experienced local transmission, evolved locally, and caused outbreaks in the cities. The intensive transportation between the two cities may have facilitated the successful and frequent exchanges of DENV strains between them.

Although Samarinda and Balikpapan are surrounded by dense rainforests, we did not detect any sylvatic DENV strain infecting our patient cohorts; all the DENVs isolated were endemic strains. Sylvatic dengue strains are known to have ecological and genetic characteristics distinct from endemic strains [14], circulate in the forest between non-human primates and arboreal *Aedes* mosquitoes [45], and have high potential for emergence as a human pathogen [46]. Although not detected in our study, it is still possible that sylvatic strains actually exist and are circulating in East Kalimantan. Recently, sylvatic DENV-1 has been isolated from a traveler visiting Brunei [37], and an ancestral strain of DENV-2 closely related to sylvatic DENV-2 was isolated from a visitor returning from Borneo [26]. More thorough and continuous surveillance, supported by comprehensive epidemiological studies, will be useful for monitoring the circulation and emergence of sylvatic DENV in Kalimantan and surrounding areas.

This study has several limitations. First, we did not assess the contribution of the dengue vector control program to the dengue epidemics. Therefore, we do not know whether a massive increase in mosquito population occurred in East Kalimantan, which ultimately facilitated the outbreaks. However, to our knowledge, no drastic measures on vector controls have been undertaken in the area. Second, the absence of prior DENV serotype and genotype data hindered our investigation of possible serotype replacement and/or cycles during the last 10 years, as serotype switching is common in countries in Southeast Asia, such as Singapore and Malaysia [31]. Third, we only managed to sequence 41 DENV isolates using RNA template directly extracted from serum samples and did not perform virus isolation/propagation in cell culture. Isolates failed to be sequenced were likely due to low virus RNA content that prohibiting the amplification and sequencing approaches. Nevertheless, our study is the first to report on the clinical, serological and virological dengue characteristics in East Kalimantan.

In conclusion, we have revealed the clinical spectrum, serology, virology, demography, and molecular genetics of DENV related to dengue outbreaks in East Kalimantan. Multiple introductions of DENV strains from surrounding regions in Indonesia have been prevalent. In addition, relatively low herd immunity was observed. Overall, these factors have contributed to the outbreaks of dengue in the area. Our data will be important for future serotype surveillance and provide information essential for future dengue mitigation in the surrounding regions.

## Additional files

Additional file 1: **Table S1** Correlation of clinical, hematological, and virological parameters with disease severity among dengue-confirmed patients in East Kalimantan. (DOCX 14 kb)

Additional file 2: **Table S2** The evolutionary parameters of DENV from East Kalimantan datasets generated by BEAST analysis. (DOCX 15 kb)

## Data Availability

Sequence data have been deposited in GenBank with accession numbers MH036375-MH036415.

## Abbreviations

DENV: Dengue virus
DF: Dengue fever
DHF: Dengue hemorrhagic fever
DSS: Dengue shock syndrome
E: Envelope protein
IR: Incidence Rate
NS1: Non structural protein 1
RT-PCR: Real-time Reverse Transcription Polymerase Chain Reaction
TMRCA: The most recent common ancestors
