# Supplementary material for: Multiple introductions of dengue virus strains contribute to dengue outbreaks in East Kalimantan, Indonesia, in 2015–2016
Source: Virol J. 2019 Jul 25;16:93. doi: 10.1186/s12985-019-1202-0 (PMC6659258; doi:10.1186/s12985-019-1202-0)
Supplement: Supplementary file 1 — Table S1 Correlation of clinical, hematological, and virological parameters with disease severity among dengue-confirmed patients in East Kalimantan. (DOCX 14 kb) [file 12985_2019_1202_MOESM1_ESM.docx]

**Table S1.** Correlation of clinical, hematological, and virological parameters with disease severity among dengue-confirmed patients in East Kalimantan.

| **Parameters** | **Disease severity** | | | ***p*-value** |
| --- | --- | --- | --- | --- |
|  | **DF (N = 35)** | **DHF (N = 95)** | **DSS (N = 2)** |  |
| Infection status | | | | 0.999^a^ |
| Primary | 21 | 58 | 1 |  |
| Secondary | 14 | 37 | 1 |  |
| DENV Serotype | | | | 0.156^b^ |
| DENV-1 | 9 | 25 | 0 |  |
| DENV-2 | 5 | 4 | 0 |  |
| DENV-3 | 16 | 48 | 2 |  |
| DENV-4 | 1 | 0 | 0 |  |
| Mix Infection | 0 | 1 | 0 |  |
| Hematology features, Median (Range) | | | |  |
| Platelet (× 10^3^/µL) | 157,000  (27,000 – 441,000) | 67,000  (4,500 – 411,000) | 54,900  (32,800 – 77,000) | **< 0.001^c^** |
| Hematocrit (%) | 38.5  (27.9 – 44.9) | 39.0  (10.8 – 52.0) | 40.25  (39.7 – 40.8) | 0.233^c^ |
| WBC (× 10^3^/µL) | 4,400  (1,700 – 12,000) | 3,950  (1,300 – 29,100) | 6,550  (6,100 – 7,000) | 0.763^c^ |

^a^ Fisher Exact Test

^b^Fisher Exact Test performed only on DENV-1, -2, and -3 vs DF and DHF

^c^ Non parametric Kruskal Wallis test calculated only for DF and DHF groups due to very low number of DSS samples
