# Supplementary material for: Multiple introductions of dengue virus strains contribute to dengue outbreaks in East Kalimantan, Indonesia, in 2015–2016
Source: Virol J. 2019 Jul 25;16:93. doi: 10.1186/s12985-019-1202-0 (PMC6659258; doi:10.1186/s12985-019-1202-0)
Supplement: Supplementary file 2 — Table S2 The evolutionary parameters of DENV from East Kalimantan datasets generated by BEAST analysis. (DOCX 15 kb) [file 12985_2019_1202_MOESM2_ESM.docx]

**Table S2.** The evolutionary parameters of DENV from East Kalimantan datasets generated by BEAST analysis.

| Parameters | DENV-1 | | DENV-2 | | DENV-3 | |
| --- | --- | --- | --- | --- | --- | --- |
|  | Median | 95% HPD | Median | 95% HPD | Median | 95% HPD |
| Tree root age (years) | 77.2 | 38.3 – 134.4 | 31.0 | 16.9 – 48.1 | 23.1 | 20.1 – 27.6 |
| Mean rate  (× 10^-4^ subs/site/year) | 8.4 | 5.5 – 11.5 | 7.7 | 5.1 – 10.6 | 9.3 | 7.6 – 11.1 |
| Coefficient of variation | 0.43 | 0.27 – 0.61 | 0.25 | 0.10 – 0.41 | 0.51 | 0.33 – 0.72 |

HPD, Highest posterior density
